# Relationship Between Mitochondrial Electron Transport Chain Dysfunction, Development, and Life Extension in Caenorhabditis elegans

**DOI:** 10.1371/journal.pbio.0050259

**Published:** 2007-10-02

**Authors:** Shane L Rea, Natascia Ventura, Thomas E Johnson

**Affiliations:** 1 Sam and Ann Barshop Institute for Longevity and Aging Studies and the Department of Physiology, University of Texas Health Science Center at San Antonio, San Antonio, Texas, United States of America; 2 Laboratory of Signal Transduction, Department of Experimental Medicine and Biochemical Sciences, University of Rome “Tor Vergata”, Rome, Italy; 3 Institute for Behavioral Genetics, University of Colorado at Boulder, Boulder, Colorado, United States of America; University of Newcastle upon Tyne, United Kingdom

## Abstract

Prior studies have shown that disruption of mitochondrial electron transport chain (ETC) function in the nematode Caenorhabditis elegans can result in life extension. Counter to these findings, many mutations that disrupt ETC function in humans are known to be pathologically life-shortening. In this study, we have undertaken the first formal investigation of the role of partial mitochondrial ETC inhibition and its contribution to the life-extension phenotype of *C*. *elegans*. We have developed a novel RNA interference (RNAi) dilution strategy to incrementally reduce the expression level of five genes encoding mitochondrial proteins in *C. elegans: atp-3, nuo-2, isp-1, cco-1,* and frataxin *(frh-1)*. We observed that each RNAi treatment led to marked alterations in multiple ETC components. Using this dilution technique, we observed a consistent, three-phase lifespan response to increasingly greater inhibition by RNAi: at low levels of inhibition, there was no response, then as inhibition increased, lifespan responded by monotonically lengthening. Finally, at the highest levels of RNAi inhibition, lifespan began to shorten. Indirect measurements of whole-animal oxidative stress showed no correlation with life extension. Instead, larval development, fertility, and adult size all became coordinately affected at the same point at which lifespan began to increase. We show that a specific signal, initiated during the L3/L4 larval stage of development, is sufficient for initiating mitochondrial dysfunction–dependent life extension in C. elegans. This stage of development is characterized by the last somatic cell divisions normally undertaken by C. elegans and also by massive mitochondrial DNA expansion. The coordinate effects of mitochondrial dysfunction on several cell cycle–dependent phenotypes, coupled with recent findings directly linking cell cycle progression with mitochondrial activity in *C. elegans,* lead us to propose that cell cycle checkpoint control plays a key role in specifying longevity of mitochondrial mutants.

## Author Summary

The worm Caenorhabditis elegans has afforded major advances in our understanding of aging, in part because a limited number of genetic pathways appear to govern aging in this organism. In this study, we explore one class of long-lived *C. elegans,* the Mit mutants, which are characterized by defective mitochondrial electron transport chain activity and, hence, ATP production. How disruption of mitochondrial function could lead to life extension has remained a mystery, especially because some of the same genes that cause life extension in worms (including *nuo-2* and *frh-1*), result in pathology in people. Here, we resolve this paradox by showing that life extension of the Mit mutants is limited to a discrete window of electron transport chain dysfunction. We show that the onset of life extension correlates with the disruption of several cell cycle–related phenomena, including larval development, adult size, and fertility and fecundity. We find no overt correlation between levels of oxidative stress and longevity. We propose that life extension in the Mit mutants is intimately connected to DNA checkpoint signaling and that the Mit mutants provide a powerful model for studying human mitochondrial disorders and aging.

## Abstract 3

Previous publications showed that the inhibition of the mitochondrial electron transport in *C. elegans* both extended and decreased lifespan. Here this paradox is resolved by showing that the fate depends on the degree of inhibition.

## Introduction

In humans, many mutations that compromise mitochondrial functionality lead to a variety of pathological, life-shortening diseases [1]. Typically, tissues with high energy requirements, such as neurons, heart, muscle, or the renal and endocrine systems, are the first to be affected. The debilitating neurodegenerative disorder Friedreich's Ataxia (FRDA), for example, is caused by the defective expression of the nuclear-encoded *FXN* gene [2]. *FXN* encodes frataxin, a 155–amino acid protein that plays an essential role in mitochondrial iron storage, Fe^2+^ detoxification, and Fe-S cluster assembly and, therefore, in the functionality of other mitochondrial proteins such as aconitase and complexes I, II, and III of the electron transport chain (ETC) [3,4].

It is particularly surprising, therefore, to learn that in *C. elegans,* a large class of mutants with disruptions (either genetic or RNA interference (RNAi)-mediated) in genes essential for the function of mitochondrial ETC—the so-called Mit (mitochondrial) mutants [5]—are long-lived. Disruption in components of all five mitochondrial ETC complexes, their intercomplex electron carriers, and the machinery necessary for their maintenance and manufacture, can lengthen life in worms from 20% to 200% [6–10]. Life extension in the Mit mutants becomes more perplexing when one considers the following: first, not all mutations that disrupt the ETC in C. elegans lead to an increase in lifespan. For example, *gas-1(fc21)* and *mev-1(kn1),* which affect complexes I and II, respectively, are both life-shortening mutations [11,12]. Second, there are at least three mitochondrial genes (*FXN, NDUSF3,* and *ANT1*) that when disrupted in humans lead to life-shortening diseases [2,13,14], yet when disrupted in *C*. *elegans,* lengthen lifespan [8,15,16]. How can loss of genes that are expected to be critical for both cellular energy production and proper mitochondrial functionality result in life extension in the *C*. *elegans* Mit mutants? Until now, most ideas have revolved around reduced mitochondrial reactive oxygen species (ROS) production with a consequent decrease in extraneous cellular damage [5,17].

The Mitochondrial Threshold Effect Theory proposes cells have the ability to counter reduction in mitochondrial ETC function up to a certain threshold but after this point, cell viability is compromised [18,19]. Indeed, mitochondrial adenosine triphosphate (ATP) production appears to be relatively robust toward interventions that impair ETC function. In rat muscle mitochondria, for example, 80%–90% of cytochrome *c* oxidase activity can be inhibited before a substantial inhibition of respiration is observed [20]. One distinction that has been largely overlooked in the study of the longevity response of the Mit mutants is the importance of reduced ETC functionality versus complete ETC disruption. Along this line, we recently proposed [21,22] that a mitochondrial threshold effect could provide insight into the mechanisms making *C*. *elegans* Mit mutants long-lived: We hypothesized that these animals operate their mitochondria at a level somewhere below normal but above that which results in pathology. It is likely that the Mit mutants manage this feat by actively invoking processes that directly compensate for their altered ETC function. We suggest that these same processes are responsible for their increased lifespan.

Here, we investigate the role of partial mitochondrial inhibition in specifying the longevity of five *C*. *elegans* Mit mutants: *atp-3, nuo-2, isp-1, cco-1,* and *frh-1* (frataxin). We employ a novel RNAi-based methodology to show that at a gene-specific level of RNAi-mediated disruption, animals respond by monotonically increasing their lifespan. Once disruption becomes too severe, however, lifespan shortens. We find there are marked alterations in multiple ETC components following each RNAi treatment, supporting the contention that compensatory processes are activated. Surprisingly, we observed no obvious connection between oxidative stress and life extension. To invoke a robust longevity response, we find that mitochondrial dysfunction must be experienced no later than the late third/early fourth larval (L) stage (L3/L4). This stage of development is characterized by the last somatic cell divisions normally undertaken by C. elegans and also by massive mitochondrial DNA expansion. Indeed, exposure of postmitotic adult worms to *atp-3* RNAi at any concentration was insufficient to invoke life extension. Recent studies have highlighted a role for mitochondrial function in the control of cell cycle progression and in the maintenance of genome integrity. We find that for all five Mit mutants that we studied, the point at which lifespan began to increase correlated with a marked reduction in the rate of four cell cycle–dependent phenotypes: postembryonic development, fecundity, fertility, and adult size. We propose that cell cycle checkpoints in proliferating somatic cells may be critical events controlling activation of compensatory mitochondrial pathways that regulate longevity in *C*. *elegans*.

## Results

### Life Extension in the *atp-3* Mit Mutant Is Limited to a Specific Window of *atp-3* mRNA Reduction

**Figure 1:**
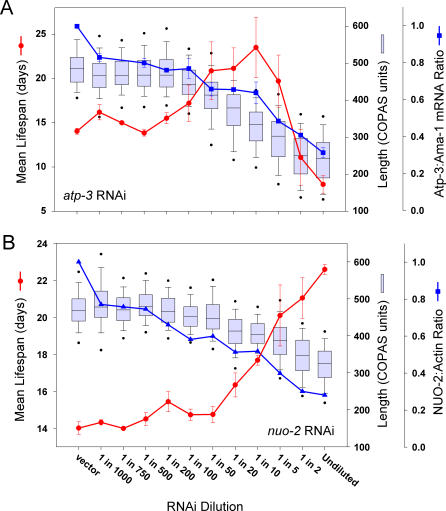
Effect of Increasing ETC Impairment on the Lifespan of *atp-3* and *nuo-2* Mit Mutants RNAi dilution series for *atp-3* (A) and *nuo-2* (B) were established using N2. Adult length (box plots in [A] and [B] show median [line], 10th–90th percentile [whiskers] and 5th–95th percentile [black dots]), mean adult longevity (± standard error of the mean [SEM], red line with circles), and total *atp-3* mRNA content (±SEM, blue line with squares), or NUO-2 protein level (blue line with triangles) were assessed for worms at each RNAi dilution (see Figure S1 for decomposition of averaged data). All animals reached adulthood except those on undiluted *atp-3* RNAi (see Figure 9). At an RNAi dilution of 1:50 for *atp-3,* and 1:20 for *nuo-2,* mean lifespan began to increase significantly (*p* < 0.05). Adult length is specified in linear COPAS units, and 500 COPAS units approximates 1 mm.

Dillin and colleagues [6], reported that the lifespan of wild-type *C*. *elegans* (N2) can be increased by approximately 30% when treated with a bacterial RNAi feeding construct against *atp-3*. This gene encodes the ATP5O/OSCP subunit of the F_1_F_o_ ATPase. Using the same RNAi construct, we found that N2 animals often arrested at the third larval stage and were actually shorter lived than vector-only–treated animals (compare vector and undiluted RNAi in Figure 1A, red line). We reasoned that life extension in worms treated with RNAi against *atp-3* might be subject to a threshold effect. To test this hypothesis, we established a simple bacterial dilution method to incrementally lower *atp-3* levels. We chose this method because of its robustness and reproducibility after first testing several other parameters known to influence RNAi expression level (IPTG, etc.) [23]. By diluting bacteria expressing *atp-3* doubled-stranded DNA (dsRNA) with bacteria containing only vector, we discovered there was indeed a limited window in which reduced *atp-3* mRNA levels (Figure 1A, blue line) led to lifespan extension (Figure 1A, red line). When *atp-3* mRNA levels were reduced to approximately 80% of normal, mean lifespan began to increase. When *atp-3* mRNA levels reached approximately 60% of normal, maximal mean life extension (on the order of 70% greater than wild type) was observed. This life extension was quite reproducible between experiments (Figure S1). When *atp-3* mRNA levels were less than 40% of normal, mean lifespan became greatly reduced.

### A Threshold Effect Also Controls Life Extension in the *nuo-2, cco-1,* and *isp-1* Mit Mutants

We next asked whether life extension in three other RNAi-defined Mit mutants was similarly constrained to occur within a discrete window of impairment. *nuo-2* encodes the NDUFS3 subunit of NADH-ubiquinone oxidoreductase [6], and an antibody against this protein is available. Using RNAi dilution, we found that NUO-2 protein levels below 60% of normal were associated with significant increases in mean adult lifespan (Figure 1B, blue and red lines, respectively). Unlike *atp-3,* lowering of NUO-2 continued to monotonically extend lifespan. At the highest concentration of *nuo-2* RNAi, protein levels were less than 30% of normal and mean lifespan was increased on average by approximately 65% (Figure S2).

**Figure 2:**
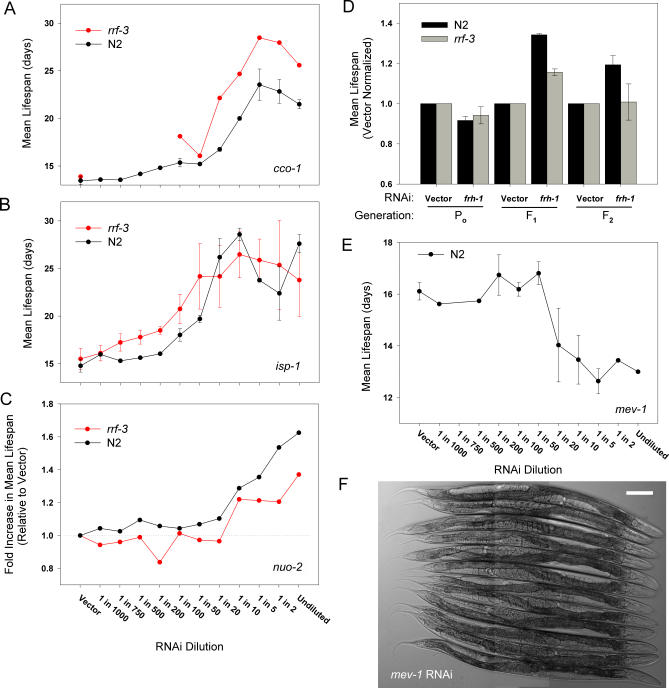
A Threshold Effect Differentially Modulates Adult Lifespan in *cco-1, isp-1, nuo-2, frh-1,* and *mev-1* Mitochondrial Mutants (A, B, and C) RNAi dilution series for *cco-1* (A), *isp-1* (B), and *nuo-2* (C) were established using N2 (black lines). Adult lifespan increases significantly (*p* < 0.05) for *cco-1, isp-1,* and *nuo-2* at RNAi dilutions of 1:200, 1:50, and 1:20, respectively. Lifespan noticeably peaks for *cco-1* and *isp-1* at dilutions of 1:10 and 1:5, respectively. The RNAi-enhancing *rrf-3(pk1426)* mutation (red lines) caused almost no alteration in the threshold for life extension within each RNAi dilution series. (Data are the average of at least two independent experiments ± SEM). (D) Wild-type (N2) and *rrf-3(pk1426)* mutant animals were cultured for three consecutive generations on bacteria expressing *frh-1* or control (vector) RNAi. Lifespan analysis of synchronous parental (P_o_), and filial (F_1_ and F_2_) populations was undertaken in duplicate for each condition (*n* = 60 worms/condition). Data for each strain, at each generation, are normalized relative to the respective vector condition. (E) A *mev-1* RNAi dilution series was established using N2 animals (*n* = 60 worms/condition). A significant life-shortening effect beginning at a dilution of 1:20 is apparent (*p* = 6.6 × 10^−13^). (F) Nomarski images of randomly selected 1-d-old animals from a *mev-1* RNAi dilution series (400×): top to bottom (target gene to empty vector ratio), 0:1, 1:1,000, 1:750, 1:500, 1:200, 1:100, 1:50, 1:20, 1:10, 1:5, 1:2, and 1:0. No size reduction at any dilution is evident) Scale bar indicates 100 μm.

*cco-1* encodes the COX5B subunit of cytochrome *c* oxidoreductase [6]. As for *atp-3* and *nuo-2,* we observed the existence of a threshold concentration after which *cco-1* RNAi initiated an increase in mean adult lifespan (Figure 2A, black line). Maximal life extension was observed at an RNAi dilution of 1:5, an increase of about 70%. Similar to *atp-3,* further increases in *cco-1* RNAi were noticeably less efficacious at increasing lifespan (undiluted *cco-1* RNAi extended mean lifespan by only approximately 60% relative to vector-only–treated N2 animals, *p* < 1 × 10^−6^).

Very few genomic mutations have been identified in C. elegans that both disrupt the mitochondrial ETC and lead to long life; one exception is *isp-1(qm150)* [7]. *isp-1* encodes the Rieske iron-sulfur protein of ubiquinol-cytochrome *c* oxidoreductase, and *qm150* is a missense point mutation. A second mutation that affects the same gene, *isp-1(gk267),* removes the entire coding region of *isp-1,* in addition to the promoter and first exonic sequence of the upstream operonic gene. *isp-1(gk267)* mutants are also long-lived (Figure S3). We tested whether simply reducing the level of *isp-1* mRNA was sufficient to prolong life. Increasingly greater amounts of *isp-1* RNAi lengthened lifespan (Figure 2B, black line). After reaching an RNAi dilution of 1:10, mean lifespan peaked and then reproducibly decreased at the following two dilutions: 1:5 and 1:2 (Figure S2). Unexpectedly, it again increased at the highest *isp-1* RNAi concentration.

Finally, we asked whether enhancing the efficacy of *nuo-2, cco-1,* and *isp-1* feeding RNAi in an *rrf-3(pk1426)* genetic background could lead to larval arrest similar to that seen using undiluted *atp-3* RNAi. The *rrf-3(pk1426)* mutant exhibits increased RNAi efficacy in several tissues, including neurons [24]. Surprisingly, we observed that the threshold RNAi concentration necessary to invoke life extension in *rrf-3(pk1426)* was essentially the same as that for N2 (Figure 2A–2C, compare red and black lines).

### Life Extension in the Frataxin Mit Mutant Is Subject to a Threshold Effect

Friedreich's Ataxia is characterized by a continuum of phenotypes that become progressively more severe as the degree of mitochondrial (frataxin) impairment increases [25]. Typically, FRDA patients present with symptoms later in life, usually by the end of childhood [21,22]. Recently, we established a *C*. *elegans* model of Friedreich's Ataxia by utilizing a bacterial feeding RNAi against the nematode ortholog of frataxin, *frh-1* [15]. Continuous feeding of *frh-1* dsRNA to N2 animals over the course of several generations led to a reduction in *frh-1* mRNA from 30% (F_1_) to 70% (F_3_). We originally observed, counter to our initial expectations, that the lifespan of animals with reduced frataxin levels was extended, not shortened, making *frh-1* a bona fide Mit mutant. Based on our above findings with *atp-3, cco-1,* and *isp-1,* we suggest that both worms and humans invoke compensatory mechanisms to counter reductions in their mitochondrial functionality following frataxin disruption: worms respond with a recordable lengthening of lifespan, whereas humans delay the pathological onset of their disease until adulthood. We propose that once a critical threshold of frataxin impairment is reached, compensatory pathways become useless in both species and lead to overt mitochondrial destruction.

One prediction of this hypothesis is that increasingly severe inhibition of *frh-1* in C. elegans should result in shortened lifespan. We tested this idea by comparing the lifespans of three consecutive generations of N2 and *rrf-3(pk1426)* animals cultured continuously in the presence of *frh-1* RNAi. As predicted, by the time the RNAi-hypersensitive *rrf-3* animals reached the F_2_ generation the life-extension phenotype of the previous F_1_ generation was lost (Figure 2D). This was not the case for N2 animals (Figure 2D), which remained long-lived relative to control animals even at the F_2_ generation, in accord with our previous observations [15]. Consistent with the increased potency of RNAi in the multigenerational, *rrf-3* mutant background, egg production in this strain became negligible (∼4%) by the F_1_ generation but was only reduced by approximately 10% in F_2_ generation N2 animals (unpublished data). Also consistent with our findings, other *C*. *elegans* models of Friedreich's Ataxia, generated using different and presumably more potent *frh-1* RNAi methodologies, exhibit a life-shortening phenotype [26,27]. We conclude that, like *atp-3,* life extension in the *frh-1* Mit mutant is subject to a threshold effect.

### Multiple Components of the ETC Are Differentially Altered Following *atp-3, nuo-2,* and *cco-1* Disruption

Metabolic control analysis has revealed that processes that significantly limit mitochondrial respiratory flux are spread across multiple sites situated both within and external to the mitochondrial ETC [28]. In addition to specific complexes of the ETC itself, substrate metabolism and transport, ATP synthesis and turnover, as well as proton leak, all differentially contribute to mitochondrial respiratory control in a tissue-dependent and a respiratory-state–dependent manner. Processes that apparently lack significant respiratory control may function as part of redundant pathways, be present in excess, or perhaps have enzyme components that normally operate under substrate concentration conditions far above their K_m_ or do so after covalent modification following a stressor. If a component normally displays marked respiratory control, then its loss might be expected to invoke significant compensatory responses in other components that also exhibit marked respiratory control, as part of a concerted cellular effort to accommodate lost metabolic potential.

To test whether loss of *atp-3, nuo-2,* or *cco-1* altered the composition of key respiratory components in *C. elegans,* we employed western analysis to monitor the expression of three mitochondrial ETC components: *nuo-2* of complex I, subunit I of complex IV, and the α-subunit of complex V. We also monitored expression of the mitochondrial adenine nucleotide transporter (ANT). All target antigens are nuclear encoded except subunit I of complex IV, which is mitochondrially encoded. Previous studies in mammalian cells have shown that the complexes containing each of these target antigens can be sites of significant respiratory control under certain conditions [28].

**Figure 3:**
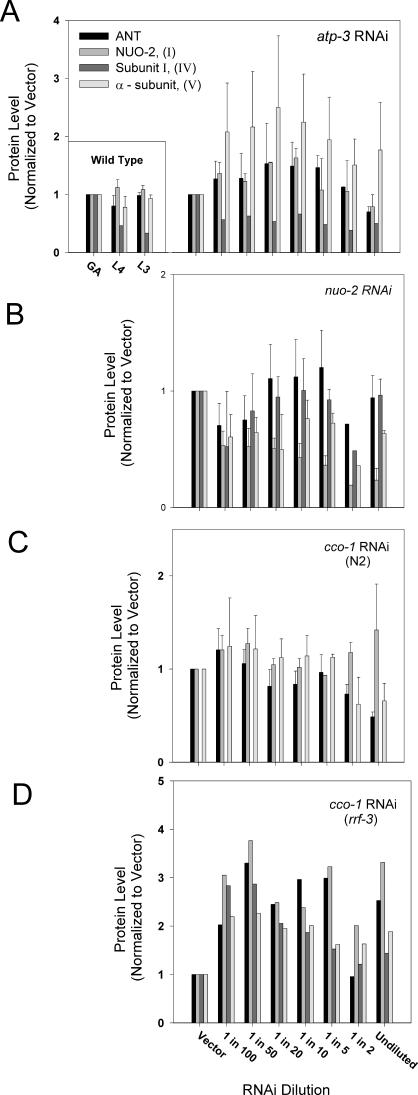
Compensatory Responses at the Level of the Mitochondrial ETC RNAi dilution series for *atp-3* (A), *nuo-2* (B), and *cco-1* (C and D) were established using wild-type (N2 [A–C]) or *rrf-3(pk1426)* mutant (D) animals (target gene to empty vector ratio: 0:1, 1:100, 1:50, 1:20, 1:10, 1:5, 1:2, and 1:0). Samples were grown until adulthood (except animals treated with undiluted *atp-3* RNAi, which remained arrested as L3 larvae), then harvested as first-day adults. Levels of four mitochondrial proteins (ANT, NUO-2 [complex I], subunit I of complex IV, and the α-subunit of complex V) were measured in whole-worm extracts using western analysis. Untreated, wild-type animals cultured on OP50 E. coli were harvested as third or fourth stage larvae (L3 and L4, respectively) as well as first-day gravid adults (GA) and then similarly subjected to western analysis ([A], inset). Differences in sample loading within each experiment were normalized against actin. Each experiment was then further normalized against the respective vector-only treatment (or relative to GA in the case of OP50-fed N2). Data in each panel represent the mean (±SEM) of at least two independent experiments (except [D], which represents a single experiment).

When N2 animals were exposed to increasing amounts of either *atp-3, nuo-2,* or *cco-1* RNAi, and whole-worm lysates were examined by western analysis, we observed substantial alterations in all four ETC-associated target antigens (Figure 3A–3C). In general, there appeared to be an initial up-regulation of each of the test antigens, which later abated at higher RNAi concentrations (although the pattern and level of the responses were RNAi dependent and RNAi concentration dependent, respectively). Interestingly, in the case of *cco-1* RNAi, we noted *nuo-2* (complex I) levels remained elevated even at the highest RNAi concentration. A similar finding was observed in the *rrf-3(pk1426)* genetic background (Figure 3D). The simultaneous alteration in multiple ETC components suggests that the changes we observed were not simply due to post-translational modification of the antibody epitopes.

In wild-type animals, gonad expansion during larval development is associated with a 30-fold increase in mitochondrial DNA number [29]. We observed a more modest 3-fold increase in mitochondrially encoded subunit I of complex IV (Figure 3A, inset: compare gravid adults with third-stage larvae [L3]). Reductions in gonad development following Mit mutant RNAi treatment (see below) may therefore, in part, explain the decreased complex IV content observed for the *atp-3* Mit mutant. If so, though, it also necessarily emphasizes the compensatory nature occurring at the level of complexes I, V, and ANT because these components are markedly increased in these animals (Figure 3A).

### Altered Levels of Oxidative Stress Do Not Appear to Underlie the Life Extension of *atp-3*

It is possible that several signaling pathways are activated in the Mit mutants following mitochondrial disruption and that one or more of these could be involved in invoking a common Mit mutant longevity response. This notion is supported by the observation that the maximum lifespan of many Mit mutants appears to be on the order of 2-fold and that ROS were recently implicated in modulating mitochondrial biogenesis [30]. In studies that will be published in full elsewhere, we have characterized the necessity of a variety of stress-activated signaling pathways to Mit mutant longevity. We briefly mention the results of one such study that is of relevance to *atp-3*.

Several chemicals, such as antimycin A and rotenone, when added at high enough doses to purified mitochondrial preparations, block electron transport and cause elevated ROS production. Indeed, the life extension phenotype of the Mit mutants can be mimicked by culturing wild-type worms in the presence of antimycin A [6]. Oxidative stress may therefore be a key factor in eliciting the Mit mutant longevity response, and furthermore, processes that normally modulate oxidative stress in worms may act to regulate Mit mutant life extension. Response to acute oxidative stress in C. elegans is controlled by the transcription factor SKN-1. This protein is the functional ortholog of the mammalian transcription factor Nrf2 [31]. In mice, Nrf2 controls the activation of some 500 antioxidant-related genes [32]. Loss of SKN-1 in worms shortens lifespan [31].

**Figure 4:**
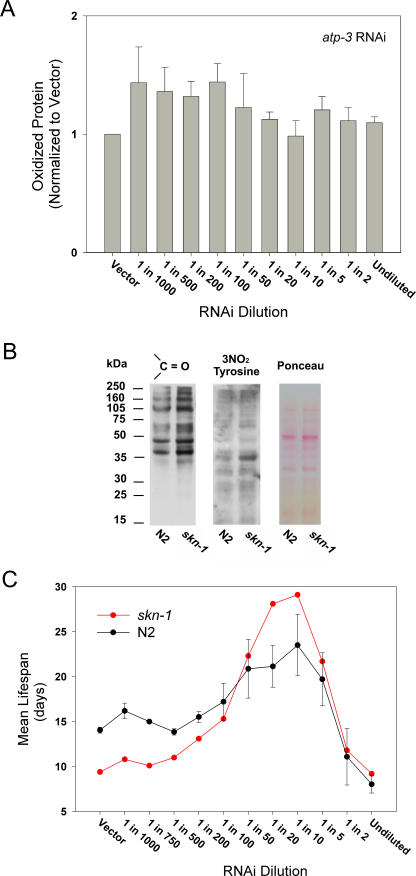
Oxidative Stress Does Not Correlate with *atp-3* Life Extension (A) Whole-worm extracts were prepared from an *atp-3* RNAi dilution series established in N2 exactly as described for Figure 3A (and including 1:1,000, 1:500, and 1:200 dilutions [*atp-3* RNAi to empty vector]). Protein carbonylation was detected using DNP chemistry coupled with western analysis. Data are presented as the mean (± SEM) of replicate experiments from a dilution series sample set normalized to vector-only treatment (following Ponceau normalization to control for differences in sample loading). (B) N2 animals were exposed to either vector or *skn-1* RNAi from the time of hatching. Levels of protein carbonylation in whole-worm, L1 extracts were detected using DNP chemistry coupled with western analysis (left panel). Samples were reprobed for 3-nitrotyrosine staining (middle panel). Equal protein loading was confirmed by Ponceau staining (right panel). A duplicate experiment showed identical results. (C) An RNAi dilution series against *atp-3* was established in both wild-type (N2, black line) and mutant *skn-1(zu67)* (red line) animals (*n* = 80/condition). *skn-1* animals were collected from 16,000 eggs as described in Materials and Methods.

To assess levels of oxidative stress in worm populations exposed to an *atp-3* RNAi dilution series, we measured protein carbonylation in whole-worm extracts (Figure 4A). We found that worms subjected to very low levels of *atp-3* RNAi (concentrations that did not result in lifespan extension) had slightly elevated protein carbonyl levels relative to control-treated animals. Across the rest of the dilution series, however, we found no correlation between degree of life extension and level of protein oxidative damage. *skn-1* also does not appear to play a role in determining *atp-3* life extension. Although animals that lacked zygotic *skn-1* displayed increased levels of oxidative stress, evident even by the L1 stage of development (Figure 4B, left panel: protein carbonylation, middle panel: protein nitrotyrosinylation), we observed neither a reduction in the degree by which treatment with *atp-3* RNAi caused life extension in a *skn-1(zu67)* mutant background relative to wild-type (N2) animals, nor a leftward shift in the life-extension peak of *skn-1* populations exposed to an *atp-3* RNAi dilution series (Figure 4C, compare red and black lines). Similar results were obtained using the *skn-1(zu135)* allele, recently shown to completely block calorie restriction–induced life extension in C. elegans [33] (unpublished data). This suggests that the longevity-inducing pathways active in the Mit mutants are fundamentally different from those activated by calorie restriction. Consistent with previous reports, we did observe that *skn-1* mutants cultured on vector-only bacteria lived, on average, 4 d fewer than N2 animals (Figure 4C). Surprisingly, the maximal increase in *skn-1* lifespan following treatment with *atp-3* RNAi (observed at a dilution of 1:10) was actually 50% greater than that observed in N2 worms, suggesting SKN-1 may in fact counter *atp-3* RNAi-mediated life extension.

### A Larval Development Signal Is Essential for *atp-3* Life Extension

**Figure 5:**
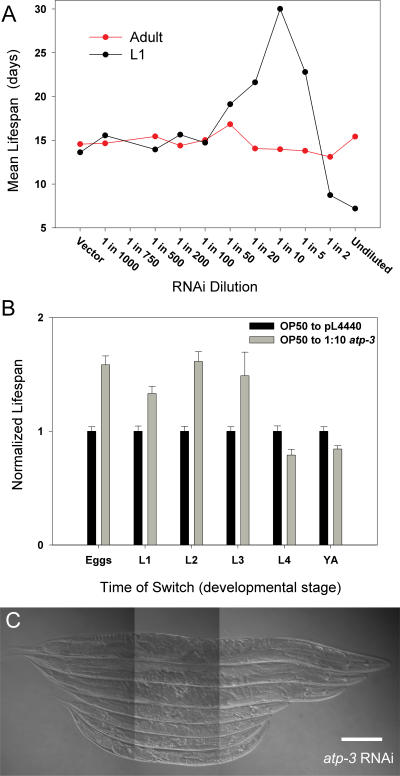
A Larval Signal Triggers *atp-3* Life Extension (A) A synchronous population of N2 was exposed to an *atp-3* RNAi dilution series either from the time of hatching (black line) or 3 d later after progression to 1-d-old gravid adulthood (red line). Life extension was evident only in animals exposed to *atp-3* RNAi from the time of hatching. (B and C) A synchronous population of N2 was cultured on a standard OP50 bacterial lawn. Worms representing each stage of development, (larval stages L1 to L4, and young adults [YA]), were transferred to vector control or 1:10 *atp-3* RNAi plates (*n* = 60/condition). The effect of *atp-3* RNAi on adult lifespan (B) and final adult size (C) is shown. Lifespan data are normalized relative to the vector control for each larval stage. Normaski image in (C) is of 2-d-old adult worms (400×): bottom to top: egg, L1, L2, L3, L4, and YA on 1:10 *atp-3* RNAi. The top animal was transferred to vector control as a YA. Scale bar indicates 100 μm.

Dillin and colleagues [6] found that addition of *atp-3* or *cyc-1* RNAi to animals during the adult stage of development is insufficient to invoke a longevity response; this was despite the fact that ATP concentrations were lowered in these animals to levels comparable to those observed in adults treated with *atp-3* or *cyc-1* RNAi from the time of hatching. Two possibilities could account for this lack of a longevity effect: an unidentified signal, process, or state is present only during larval development and is necessary to translate the reduction in ATP concentrations into a longevity response. Alternatively, adult animals may experience an altered sensitivity to RNAi, effectively shifting their RNAi dose-response curve. We sought to distinguish these possibilities by exposing first-day N2 adults to a full *atp-3* RNAi dilution series and monitoring its effect on remaining longevity. We saw no increase in lifespan at any RNAi concentration tested (Figure 5A), despite a dramatic absence of fertile egg production by adult animals exposed to undiluted *atp-3* RNAi (unpublished data). N2 hatchlings exposed to an identical *atp-3* RNAi dilution series exhibited a robust lifespan extension effect (Figure 5A). We confirm that an unidentified, larval developmental event is necessary for *atp-3* RNAi-mediated life extension.

We next sought to determine the last larval stage at which animals remained sensitive to this developmentally specified, life-extension event. Synchronous populations of N2 animals representing each stage of larval development were transferred to vector control or *atp-3* RNAi (1:10 dilution), and lifespan was recorded. Figure 5B reveals that the L3 stage of larval development represents the limiting stage at which *atp-3* RNAi is able to induce life extension. The appearance of life extension was strongly associated with adult size reduction (Figure 5C). Because our *atp-3* RNAi construct took approximately 12 h to become phenotypically efficacious (see below and Table S1), we conclude that the L3/early L4 stage of development represents the limiting period for specifying life extension following mitochondrial disruption in *C*. *elegans*.

### Adult Size, Postembryonic Developmental Rate, and Fecundity and Fertility Are Coordinately Retarded by Increasing Mit Mutant RNAi Concentration

The early stages of L4 development represent the time when the last somatic cell divisions occur in *C*. *elegans*. It is possible that cell cycle disruption during development is required to signal life extension in the Mit mutants, explaining why postmitotic adults are refractory to the effects of *atp-3* (Figure 5A) and *cyc-1* RNAi [6]. In worms, mitochondrial activity has been intimately connected with cell cycle progression ([34] and see Discussion). We therefore systematically investigated the effect of *atp-3, nuo-2, isp-1,* and *cco-1* RNAi on four phenotypes dependent upon cell cycle progression: adult size [35], larval developmental rate [36], and fecundity (egg production) and fertility (F1 embryonic viability) [37].

**Figure 6:**
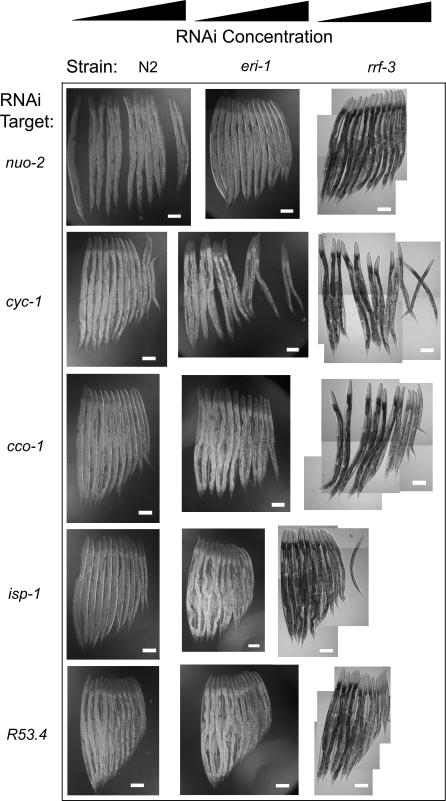
Adult Size Decreases with Increasing Mit Mutant RNAi Concentration RNAi dilution series for *nuo-2, cyc-1, cco-1, isp-1,* and *R53.4* were established using N2 (left column), *eri-1(mg366)* (middle column), and *rrf-3(pk1426)* (right column) animals. When vector-only treated animals became 1-d-old gravid adults, individual worms were randomly selected from each RNAi dilution and mounted for Normaski imaging (400×): left to right (target gene to empty vector ratio), 0:1, 1:1,000, 1:750, 1:500, 1:200, 1:100, 1:50, 1:20, 1:10, 1:5, 1:2, and 1:0. Scale bars indicate 100 μm.

We observed a striking phenotype when exposing N2 animals to increasing concentrations of each RNAi: adults became increasingly smaller at maturity (Figures 1 and 6). We found that the *onset* of adult size reduction strongly correlated with the *onset* of life extension in all four dilution series. Interestingly, absolute size reduction was not a consistent predictor of the amount of life extension, since severe size reduction was actually associated with shortened life. We quantitated the effect of *atp-3* and *nuo-2* RNAi dilutions on adult size using a mechanical worm sorter (Figure 1A and 1B, box plots). Animals were sized at the same chronological age and were adults except those cultured on undiluted *atp-3* RNAi, which were instead arrested L3 larvae (see below). Similar effects on adult size were also observed using RNAi against *cco-1* and *isp-1* (Figure 6) and several other Mit mutant genes (Figure S4). Surprisingly, both *rrf-3(pk1426)* and *eri-1(mg366),* another genetic mutant exhibiting hypersensitivity to RNAi, displayed no enhancement relative to N2 animals in their sensitivity to size reduction. Together, our findings imply there may be a common mechanism acting to control both size reduction and longevity enhancement in the Mit mutants.

**Figure 7:**
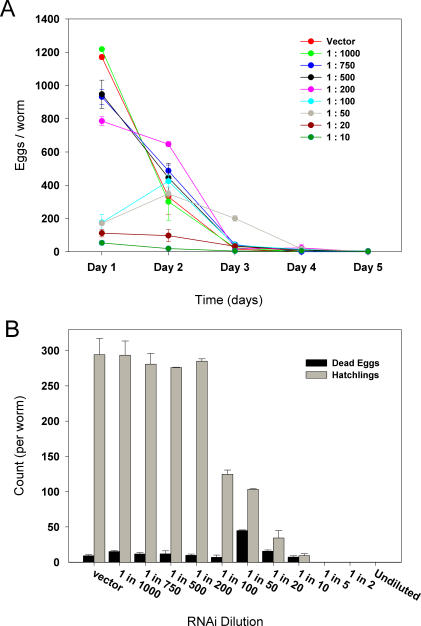
Egg Production Rate, Fecundity, and Fertility Decrease with Increasing *atp-3* RNAi Concentration An RNAi dilution series for *atp-3* was established using N2. Egg production rate (A), and fecundity and fertility (B) were measured as described (Materials and Methods). Day 1 corresponds to the start of egg laying for each dilution. In (A), animals treated with 1:5, 1:2, and undiluted *atp-3* RNAi failed to lay eggs and have been omitted.

**Figure 8:**
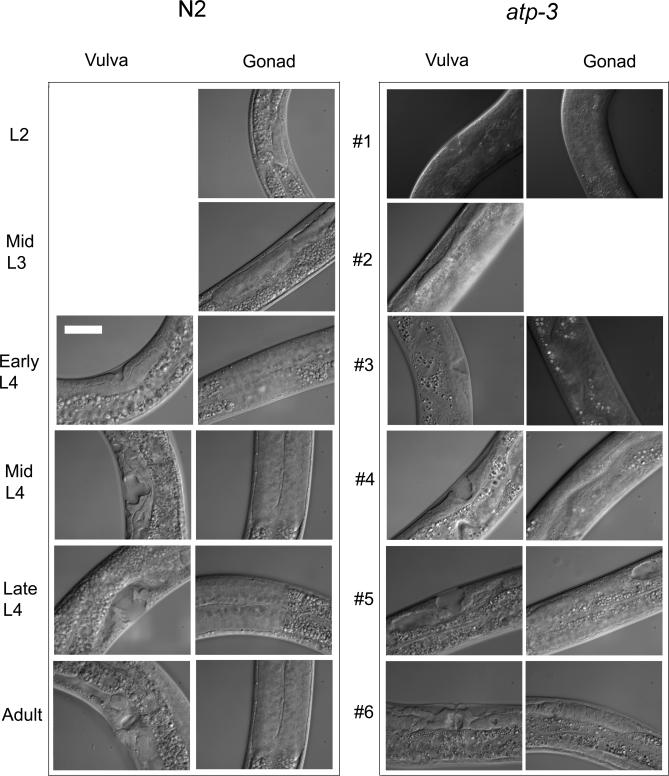
Rate of Larval Development Decreases with Increasing *atp-3* RNAi Concentration N2 animals exposed to undiluted *atp-3* RNAi (right panels) from the time of hatching were collected for Normaski imaging (1000×), 4 d later (worm #1) and 6 d later (worms #2–6) . Shown is the extent of vulval and gonadal development in selected individuals. For comparative purposes, N2 animals (left panels) were cultured on control bacteria and collected during various stages of their development. Scale bar applies to all panels, and indicates 25 μm.

At the RNAi concentrations of *atp-3, nuo-2, cco-1,* and *isp-1* that initiated a decrease in adult size (and a significant increase in lifespan), the rate of postembryonic development, adult egg production (fecundity), and F_1_ embryo viability (adult fertility) also all became retarded. The relative severity of this effect differed between each RNAi, but it uniformly increased with increasing RNAi concentration. *atp-3* and *nuo-2* represent the phenotypic extremes that we observed (quantitative data for *atp-3* are provided in Figure 7A and 7B and Table S1). N2 animals fed different dilutions of *atp-3* RNAi exhibited either almost permanent L3 arrest (undiluted *atp-3,* Figure 8), temporary late L4 arrest with slow maturation into sterile adulthood (1:5), or a markedly slowed progression to adulthood with reduced progeny production (1:10, 1:20, and 1:50). Dilution of *nuo-2* RNAi resulted in a very different set of phenotypes. Undiluted *nuo-2* caused a 12-h delay in the time it took for N2 animals to become egg-laying adults, and it led to about a 50% reduction in egg number, but only a marginal decrease in the viability of these eggs. All *nuo-2* effects became negligible at dilutions greater than 1:20 (unpublished data). We conclude that increasingly severe reduction of mitochondrial ETC function during larval development delays (and eventually arrests) larval development, reduces adult size, diminishes fecundity, and ultimately causes complete sterility. Lifespan control appears therefore to be coupled to several processes that share intimate links with cell cycle progression.

**Figure 9:**
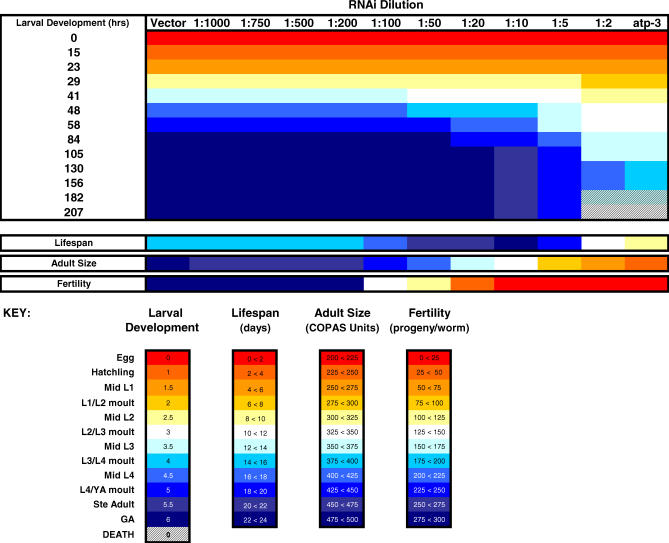
Concordance of the Mit Phenotypes Four phenotypes (top to bottom panels: mean larval developmental rate, mean lifespan, mean adult size, and mean fertility) were scored across an *atp-3* RNAi dilution series. Data are derived from Figures 1 and 7 and Table S1 and is tabulated in a color-coded format (key). All four phenotypes become disrupted concordantly. At an RNAi dilution of 1:100 mean lifespan, mean adult size and mean fertility became recognizably different from vector-treated animals. By 1:50, mean larval development (assayed using a dissecting microscope and not high-power optics) became noticeably retarded. Larval development (top panel) was quantitated as follows: 30 freshly laid eggs were placed onto each of the indicated *atp-3* RNAi dilutions (top row). At the listed times (left column), the stage of development of each hatchling was scored. An average developmental stage at each time interval was calculated by assigning each larval stage a number (shown in key) and then taking the average value for each 30-worm population.

### Absence of a Life-Extending Effect in a *mev-1* RNAi Dilution Series

None of the known Mit mutants involve disruption of complex II (Table S2). This could simply be due to the fact that there are only four subunits in this complex, and the vagaries of both genetic and large-scale RNAi screening may simply have missed the relevant targets. More refined studies using RNAi specifically targeting each of the four subunits still revealed no life-extension effect [38]. Redundancy of function might therefore be partly responsible since the succinate dehydrogenase subunit of complex II has a very closely related paralog [39]. Alternatively, given our findings regarding the threshold effect for each of the Mit mutant genes we have tested so far, it is also possible that previous screens may have missed complex II components because they failed to attain some critical level of complex II inhibition. We decided to test this latter idea using a *mev-1* RNAi dilution series. *mev-1* encodes the cytochrome *b* subunit of succinate-ubiquinone oxidoreductase (complex II) and is involved in shuttling electrons into the mitochondrial inner membrane and onto ubiquinone. We established an RNAi dilution series against *mev-1* in N2 animals and recorded both lifespan and adult size data (Figure 2E and 2F, respectively). Contrary to our predictions, we observed no life-enhancing effect at any of the tested RNAi concentrations (Figure 2E). Surprisingly, we did see a dramatic 4-d reduction in mean adult lifespan at dilutions of 1:20 and below. Interestingly, adult size at these dilutions was also affected, but this effect only became evident on the second day of adulthood (compare 1-d-old adults shown in Figure 2F with older animals from the same experiment shown in Figure S5). Animals from all dilutions seemed to be comparably sized during larval development. The efficacy of our *mev-1* RNAi was underscored by the fact that, at RNAi dilutions of 1:20 and less, adult animals laid an increasingly larger fraction of eggs that never hatched (Figure S5). Finally, we observed a very similar phenotypic effect on N2 adult size when targeting *tag-55,* the Fe-S subunit of complex II (Figure S4).

## Discussion

The Mit mutants of *C*. *elegans* have posed an unanswered riddle: how can disruption of the mitochondrial ETC cause life extension? In the present study, we have shown in fact that only under conditions of partial ETC disruption is life extension observed. As might be expected, more-severe disruption of the ETC shortens life. In this study, we have also confirmed previous results showing that adult *C*. *elegans* are refractory to the life-extending effects of *atp-3* RNAi, and we now reveal there is a minimal L3/early L4 larval requirement for lifespan extension. Furthermore, our findings suggest that increased levels of oxidative stress are evidently not correlated with Mit mutant life extension. Finally, we have shown that several phenotypes collectively associated with cell cycle progression in *C*. *elegans* become coordinately disrupted in the Mit mutants. Interestingly, these phenotypes track with the appearance of life extension.

### Defining the Mit Phenotype

Several features collectively define the Mit phenotype: these include extended longevity, reduced adult size, slowed larval development, decreased fecundity, and decreased fertility. In all five of the mitochondrial mutants that we studied (namely, *atp-3, nuo-2, cco-1, isp-1,* and *frh-1*), we found that disruption of growth, retarded larval and gonad development, as well as reduced fertility and fecundity, all occurred coincidently with the appearance of life extension. The concordance of these phenotypes is illustrated quite strikingly in Figure 9, in which we have provided a quantitative measure of each phenotype across an *atp-3* RNAi dilution series. The Mit phenotype also exhibited a graded response in that all phenotypes became increasingly more severely disrupted up to the point at which lifespan became maximal. Our findings are consistent with a model in which all five processes are dependent on the same limiting function or functions. Shortly, we will propose that cell cycle checkpoint signaling, activated following mitochondrial dysfunction, is at the root of the Mit phenotype.

### Recent Models for Why the Mit Mutants Are Long-Lived

Several models have been put forward as to why the Mit mutants are long-lived. One of the first proposed that a general reduction in metabolism may be sufficient to account for life extension. It was argued that simply living slower would mean that the time taken to reach some critical life-ending event would take longer [40]. In a variation of this “Rate-of-Living” model, Sedensky and Morgan [17] proposed that reduced ETC function might account for life extension of the Mit mutants when accompanied by reduced ROS production. Third, we have previously proposed that Mit mutants may be long-lived by virtue of the use of alternate metabolic pathways, many of which bypass the mitochondrion altogether, and as a consequence, reduce mitochondrial ROS production [5,39].

Our present findings argue against the first model because it predicts that animals that developed the slowest, such as those cultured on undiluted *atp-3* RNAi, should have lived the longest. The counterargument, that oxidative stress could be masking a true longevity effect at high *atp-3* concentrations, is significantly weakened by our inability to detect increased levels of protein oxidation in these worms. More generally, it is hard to imagine how metabolic rates can be slowed down in a poikilothermic organism. Recent studies in Escherichia coli show that disruption of central carbon metabolism (similar to what must be happening in the Mit mutants) leads to localized metabolic alterations that act to maintain cellular metabolites in a near-normal state [41]. Additional studies have discounted model 1 [42]. Model 2 is weakened by results derived using purified mitochondria showing that ROS production often becomes elevated when ETC flux is blocked by complex-specific inhibitors. This effect is of course dependent upon the exact site of inhibition within the ETC, but the seemingly indiscriminate nature of ETC targets within the set of 38 known Mit mutants (listed in Table S2) weighs against this model. Model 3 was not further tested by our current set of experiments, although our findings do not exclude activation of alternative metabolic pathways. Indeed, such processes may well comprise some of the compensatory pathways possibly activated in the Mit mutants to help supplement cellular ATP production.

### Oxidative Stress and Mit Mutant Longevity

Many studies suggest a connection between increased oxidative stress and aging (for a comprehensive review, see [43]). For this reason, reduced ROS production has featured prominently in several models aimed at explaining why Mit mutants are long-lived, as described above. Our current findings suggest, however, that there is almost no alteration in the degree of oxidative damage occurring in *atp-3* Mit mutants. Furthermore, removal of SKN-1, the key transcription factor necessary for counteracting oxidative challenges in worms [44,45] (and unpublished data) appeared to have no effect on the ability of *atp-3* RNAi to induce life extension. Our findings therefore question the role of oxidative stress in inducing Mit mutant longevity.

There are, nonetheless, many caveats in interpreting our oxidative stress findings: first, it is possible that oxidative stress in worms can be mitigated by a SKN-1–independent system. Second, measurement of protein carbonylation may not provide an accurate reflection of the kind of oxidative damage occurring in the Mit mutants. It is hard, though, to reconcile how oxidative species could differentially avoid protein targets. Third, analysis of whole-worm lysates might lack sufficient resolving power to discriminate subtle oxidative differences across our *atp-3* dilution series. More refined studies using purified mitochondria will be required to resolve this issue. Finally, regarding SKN-1, one might argue that maternally contributed SKN-1 simply masked the true effect of loss of zygotic SKN-1 on Mit mutant longevity. This idea seems unlikely, however, because elevated oxidative stress was apparent even in the L1 larvae of *skn-1* animals.

### Reduction of Mitochondrial Function beyond a Certain Threshold Triggers Increased Longevity

In the present study, we employed a simple RNAi methodology to incrementally disrupt ETC function in worms. Although we did not formally record reduced flux through the ETC, or through specific RNAi-targeted complexes, we note that a recent study using RNAi against one of our target antigens (frataxin), detected reduced whole-animal oxygen consumption [27]. In addition, Dillin and colleagues [6] have shown that RNAi-mediated reduction of *atp-3,* using the same bacterial feeding construct employed in the present study, resulted in decreased ATP production. On the basis of these findings, we think it is a reasonable first assumption to conclude that ETC activity was reduced across each of the RNAi dilution series we studied. Indirect support for this idea comes from our observation that multiple non–RNAi-targeted ETC components were altered in *atp-3, nuo-2,* and *cco-1* Mit mutants. RNAi is a largely cell-indiscriminant methodology. Because the mode by which different cell types invoke respiratory control may vary, and because the kinds of compensatory processes invoked by cells to supplement lost ETC functionality also may vary, what we observed by western analysis likely represents an average of different cellular responses.

With these caveats in mind, we have observed that quantitative reduction in the expression of either *atp-3* or *nuo-2* at the mRNA and protein level, respectively, resulted in life extension only within a discrete window of ETC disruption. In the case of *atp-3,* once disruption became too severe, deleterious effects were observed. Similar effects were qualitatively observed for *isp-1, cco-1,* and *frh-1* (see also Ventura et al., 2005 [15]). Partial ETC inactivation appears, therefore, to be essential for Mit mutant life extension. We presume that the processes initially evoked to counteract low levels of mitochondrial ETC disruption resulted in *atp-3, nuo-2, isp-1, cco-1,* and *frh-1* life extension, but that these processes were incapable of maintaining vitality at higher levels of mitochondrial dysfunction. This model suggests that perhaps all of the known Mit mutations cause a reduction, rather than complete loss, of mitochondrial ETC function. Indeed, most Mit mutants have been defined by RNAi, which is a knock-down rather than a knock-out technology. Our findings provide an explanation for why many of the large-scale RNAi screens in *C*. *elegans* for enhanced longevity [6,8,9,46] did not identify the same Mit mutants: differences in RNAi efficacy presumably placed different Mit mutants at different points along their mean life-extension curves. Consistent with this idea, only one gene, *cco-1,* was reproducibly identified in four independent RNAi longevity screens (see Table S2). Other reasons could also account for the variation in the genes identified in each of the large-scale RNAi screens, including differences in library representation, the strains used for screening, reagent concentrations, and technical variability. Nonetheless, our data reveal that minor changes in mRNA can lead to dramatic phenotypic differences.

### The Role of Larval Development and Cell Cycle Progression in Mit Mutant Longevity Specification

Several pieces of evidence lead us to now propose that disruption to normal somatic cell cycle progression during larval development is sufficient to induce the Mit phenotype in *C*. *elegans*.

Larval development in *C*. *elegans* proceeds via a series of four molts. After embryogenesis, animals hatch containing 558 cells, and approximately 10% of these cells undergo further divisions to result in an adult containing 959 somatic nuclei [47]. Gonadogenesis continues throughout each of the four larval stages, but it accelerates greatly at the L3 stage. The last somatic divisions, those necessary for generation of the somatic gonad, are completed by the early stages of L4. In the present study, we have shown that the L3/early L4 stage of development is the last stage at which *atp-3* RNAi can be added to cause life extension. Furthermore, we also show that four phenotypes intimately linked to cell cycle progression, namely, adult growth (see below), larval development rate [36], gonadogenesis, and fertility [37], all became coincidently disrupted with the appearance of life extension. There appeared to be a graded response in that each of these four phenotypes became increasingly more severely disrupted up until the point at which lifespan became maximal. The L3 stage of development is a critical period in the development of C. elegans. This is the time when maternally provided cyclin E runs out [36] and when mitochondrial DNA (mtDNA) undergoes massive numerical expansion [29]. Interestingly, almost all null mutants of ETC components that we know of, including *atp-2(ua2), frh-1(ok610),* and *isp-1(gk267),* never progress beyond the L3 stage of development [16,48]. Size control in C. elegans occurs as a consequence of both cell division and cell growth [49,50]. In the adult, endoreduplication in the hypodermis and intestine also contributes to growth. This process of DNA re-replication without nuclear division occurs in a TGFβ and cyclin E–dependent manner [51].

Curtis and colleagues [52] recently showed that genetic removal of adenosine monophosphate (AMP)-activated protein kinase (AMPK) almost completely abolished the life extension of both *isp-1(qm150)* and *clk-1(qm30)*. Reduced ATP levels therefore play a major role in specifying Mit mutant longevity. AMPK monitors cellular energy status by sensing the AMP:ATP ratio. When AMP levels are high, AMPK switches on catabolic processes that generate ATP while simultaneously switching off ATP-consuming processes [53]. Of particular relevance, AMPK was recently shown to control activation of a cell cycle checkpoint that governed passage through the G_1_/S boundary of primary mouse embryonic fibroblasts [54]. This signal was found to be mediated by activation of the p53 tumor suppressor protein. An almost identical finding was discovered in Drosophila melanogaster [55]. Eye tissue containing one of four different recessive lethal mitochondrial mutations (two in mitochondrial ETC components and two in mitochondrial ribosomal proteins) arrested at a G_1_/S checkpoint in an AMPK- and p53-dependent manner. Cyclin E was shown to be posttranslationally down-regulated in these cells and to reside downstream of both AMPK and p53. *Cep-1* is the worm homolog of p53 [56]. As might be predicted, we have now found a role for this protein in defining the Mit phenotype of C. elegans (unpublished data).

Of particular relevance to the current study, in a recent genome-wide RNAi screen designed to isolate genes required for the first two rounds of embryonic cell division in *C*. *elegans,* 661 genes were identified that were further subgrouped into 23 phenotypic classes based on the type of developmental disruption they caused [34]. Intriguingly, 48 of the 661 identified genes encode mitochondrial proteins (50% more than expected by chance [57], Table S2), most of which belonged to a subclass defined by a marked retardation in the pace of development during the first 30 min of embryogenesis. Nineteen of the 48 mitochondrial genes encoded direct components of the ETC (over four times more than expected by chance). Of particular interest, 13 of these 48 mitochondrial genes are known to result in a bona fide Mit phenotype when disrupted by RNAi (Table 1: *atp-2, atp-3, atp-4, aco-2, asb-1/2, asg-1/2, hsp-6, nuo-2, nuo-4, lrs-2, D2030.4, T20H4.5,* and *T27E9*.*1*). These findings therefore provide additional support for the idea that disruption of the normal division of somatic cells is primarily involved in triggering the longevity response of the Mit mutants.

**Table 1:**
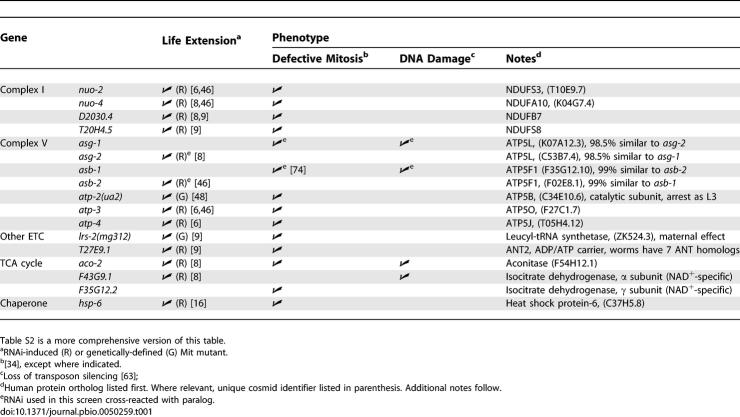
Overlapping Roles of Genes Associated with Mit Mutant Longevity, Embryonic Mitosis, and DNA Integrity

Cell cycle arrest is usually only invoked in dividing cells to either avoid DNA damage when ATP supplies are low, or to repair already damaged DNA [58]. In the Mit mutants, we propose that both processes act to stall the cell cycle, delay larval development, and in turn, signal life extension. Studies using the yeast Saccharomyces cerevisiae show that mitochondrial ETC dysfunction, resulting from mtDNA deletion, chemical inhibition, or *frh-1* mutation, leads to an elevation in the mutational rate of nuclear DNA [59,60]. Similar findings have been recorded in mouse blastocysts [61] and human fibroblasts [62]. Data directly supporting the notion that elevated DNA damage occurs in the *C*. *elegans* Mit mutants come from the work of Vastenhouw and colleagues (2003) [63]. These researchers undertook a genome-wide RNAi screen for genes that when silenced, caused transposition of the Tc1 transposon in the germline of *C*. *elegans*. Normally, these motile elements are kept inactive in an ATP-dependent manner to avoid germline mutations [64]. Of the 27 genes they identified, ten encoded mitochondrial components (Table S2). Four of these genes are known to result in a bona fide Mit phenotype when disrupted by RNAi (Table 1: *aco-2, asb-1/2, asg-1/2,* and *F43G9.1*). Interestingly, these latter four genes were also identified in the embryonic cell cycle disruption screen described earlier [34]. How DNA damage might confer life extension remains unclear, but disruptions to components of conserved DNA damage responses are already known to increase *C*. *elegans* lifespan [65–67]. Interestingly, the long-lived *clk-2* mutant which displays many phenotypic characteristics of the Mit mutants (and was isolated in the same genetic screen that identified *clk-1*) is in fact a DNA damage-response protein [68]. A recent study has now shown that the developmental delay observed in *clk-2* mutants is dependent upon p53/*cep-1* signaling [69].

### A New Model for Life Extension in the Mit Mutants

We propose a new model explaining the long life of the Mit mutants. Specifically, we suggest that (1) compromised mitochondrial ETC function reduces ATP levels during larval development, alters both the concentration and ratio of nucleotides, and increases cellular oxidative stress. As a consequence, (2) DNA synthesis is disrupted, leading to both nuclear and mtDNA damage, stalling of the cell cycle, and in turn, activating nuclear checkpoint signaling. We posit that (3) multiple stress-response pathways that increase nuclear and mtDNA repair, and compensate for reduced mitochondrial efficiency, are induced. These pathways turn on alternative ATP production routes and/or redistribute respiratory control to different mitochondrial components, and activate antioxidant mechanisms. In essence, a metabolic profile is adopted that is designed for organismal perdurance. We predict that steps (1) and (2) are tightly linked, because only developing larvae with their mitotic soma can respond to compromised ETC functionality.

### Why Do All Mitochondrial Mutations Not Extend Lifespan?

Not all mutations in mitochondrial components result in life extension. We were unable to reveal a Mit phenotype when we lowered the levels of wild-type *mev-1* by RNAi at any concentration. The *mev-1(kn1)* allele encodes a G71E missense mutation in cytochrome *b* that results in a dysfunctional ubiquinone binding site. As a consequence, not only is the rate of oxidative phosphorylation decreased in this mutant, but there is a concomitant increase in both superoxide anion production and supernumerary apoptotic events that, together, act to shorten lifespan [70]. To what extent our *mev-1* RNAi phenocopied the *mev-1(kn1)* point mutation is unclear. One possibility is that a maternal contribution of *mev-1* RNA may have masked the life-extension phenotype; maternally contributed product is already known to mask longevity in one other Mit mutant, *clk-1* [10]. A maternal effect could, incidentally, also provide a simple explanation for why two strains that normally display hypersensitivity to RNAi, namely *rrf-3(pk1426)* and *eri-1(mg366),* did not exhibit any enhancement of their Mit phenotype relative to N2 animals in our present study. Specifically, no amount of mRNA reduction in larvae by RNAi would affect the levels of protein already present from the time of hatching. An alternate explanation, encompassing tissue-specific roles for each Mit mutation coupled with the absence of a differential RNAi-enhancing affect in these tissues by *rrf-3(pk1426)* and *eri-1(mg366),* cannot be excluded at this stage.

### Human Mitochondrial-Associated Diseases and Mitochondrial Threshold Effects

Human Mitochondrial-Associated Diseases (HMADs) are characterized by dramatic variability in their phenotypic presentation [1]. Indeed, mutations in the same gene can present with different pathological outcomes, whereas mutations in different genes can manifest the same pathology. Cell types differ in the relative amount of ETC disruption they can tolerate and in their ability to counteract such deficits. Variations in mitochondrial DNA copy number, transcriptional and translational efficacy, mitochondrial biogenesis, and posttranslational alteration to components of the ETC, all contribute to the heterogeneity in cellular responses [19]. These facts and others led to the proposal of the Mitochondrial Threshold Effect Theory: the notion that cells can variably respond to the physiological deficit of impaired ETC by invoking a series of counteractive pathways, but beyond a certain threshold of mitochondrial dysfunction, even these processes become unable to compensate, and viability is irrecoverably compromised [18,19].

We have previously proposed that the processes that act in the Mit mutants to offset ETC dysfunction and increase lifespan may be the same ones that act to delay clinical appearance of many mitochondrial disorders [16,21]. In the present study, we have shown that mild reduction of frataxin in worms results in life extension, but more severe reduction actually shortens lifespan. These findings now provide support for our contention that the *C*. *elegans* Mit mutants offer a new model for the investigation of human mitochondrial-associated disorders. Several other genes associated with human mitochondrial-associated diseases have Mit mutant counterparts [16], opening up the possibility of developing new genetic models to study these conditions.

### Conclusion

In this study, we have shown that partial disruption of the ETC causes an increase in *C*. *elegans* lifespan and retardation in growth, development, and gonad maturation. Mild mitochondrial disruption may lead to transient DNA (nuclear and mitochondrial) damage and cell cycle retardation, leading to activation of life-prolonging, compensatory mechanisms. Our simple method of using RNAi dilution to incrementally lower ETC functionality offers the potential to precisely define both the mitochondrial parameters and the signaling events that are necessary to confer maximal longevity to C. elegans. This method also provides the possibility of testing these same parameters in mammalian cells.

## Materials and Methods

### Nematode maintenance.

Standard nematode culturing techniques were employed [71]. The following strains were utilized: N2 (wild type), GR1373 (*eri-1(mg366)* IV), NL2099 (*rrf-3(pk1426)* II), VC520 (*isp-1(gk267)IV/nT1(qIs51)* (IV;V)), EU1 (*skn-1(zu67) IV/nT1[unc-?(n754) let-?]*(IV;V)), EU31 (*skn-1(zu135) IV/nT1[unc-?(n754) let-?]*(IV;V)), and CL691 (*dvIs19[pAF15(gst-4::GFP::NLS)* III); *skn-1(zu67)* IV/*nT1*[*unc^D^-?(n754); let-?*](IV;V)). CL691 was constructed from a double cross between CL2166 (*dvIs19[pAF15(gst-4::GFP::NLS)]* III) and EU1 (*skn-1(zu67) IV/nT1[unc-?(n754) let-?]*(IV;V)). *skn-1(zu67* and *zu135)* are maternal effect lethal.

### RNAi dilution series.

*atp-3* (F27C1.7)*, nuo-2* (T10E9.7), *cco-1*(F26E4.9), *cyc-1*(C54G4.8), *mev-1* (T07C4.7), R53.4, D2030.4, *tag-55* (F42A8.2), F26E4.6, and W09C5.8 bacterial feeding RNAi constructs were retrieved from the Ahringer C. elegans RNAi library [72]. *isp-1* (F42G8.12) RNAi was generated ab initio (corresponding to genomic fragment IV:8133946..8136020). For dilution series, cultures of HT115(DE3) containing empty vector (pL4440) or each gene of interest were prepared in LB broth containing 100 μg/ml ampicillin and 5 μg/ml tetracycline and grown overnight at 37 °C. Optical density at 590 nm (OD_590_) values were adjusted to 0.9 and bacteria mixed in the following target gene to pL4440 ratios: 0:1, 1:1,000, 1:750, 1:500, 1:200, 1:100, 1:50, 1:20, 1:10, 1:5, 1:2, and 1:0. For each mix, 250 μl was added to a 6-cm RNAi plate (NGM agar + 1 mM IPTG, 100 μg/ml ampicillin, and 5 μg/ml tetracycline) and grown overnight at 23 °C. Eighty N2, *eri-1,* or *rrf-3* eggs (or 1-d-old gravid adults for the *atp-3* dilution series shown in Figure 5B) from a 4-h–limited lay were transferred from standard OP50 culture plates to each type of RNAi plate and allowed to develop at 20 °C.

### Consecutive generational RNAi.

For studies involving frataxin, N2, and *rrf-3(pk1426),* animals were cultured on pL4440-containing or *frh-1* RNAi-producing HT115(DE3) bacteria for three consecutive generations as described previously [15]. Longevity assays were initiated for each generation when vector-only–treated animals first reached adulthood.

### Lifespan analysis.

Lifespan was recorded at 20 °C using synchronous populations of 60–80 animals per RNAi dilution (or at each generation for *frh-1* RNAi studies). Since there was typically a delay in the time it took animals on the lowest dilutions to reach adulthood (usually 12–24 h), lifespan analyses began when vector-only–treated animals first reached adulthood. Data were analyzed using the log-rank test as previously described [73]. *Skn-1(zu67)* homozygous animals were isolated from CL691. A 16-fold excess of synchronously laid eggs from balanced hermaphrodites was transferred to each RNAi dilution plate, then allowed to reach adulthood. From this population, 60–80 *skn-1* animals were isolated, and their survival was recorded.

### *atp-3* fecundity, fertility, and developmental analysis.

RNAi dilution series against *atp-3* were established using N2 animals. For measurement of fecundity and fertility, synchronous populations of eggs were placed onto each dilution plate, allowed to mature, and then on the first day of adulthood, replicate batches of five animals were removed and followed over a period of 5 d. Total egg number, as well as the total number of hatched progeny (scored after 2 d), was recorded for each day. Animals that crawled off plate, bagged, or exploded were replaced with an age-matched animal grown in parallel. For the analysis of larval development, populations of 30 synchronous animals were followed from the time of hatching. Vulval and gonadal developmental was used to score developmental stage.

### Quantitative-PCR.

Quantitation of *atp-3* mRNA was undertaken in triplicate using *ama-1* (F36A4.7) ratiometric quantitative-PCR (Q-PCR) (ABI Prism 7000; Applied Biosystems, https://www2.appliedbiosystems.com). mRNA was extracted from 5,000–15,000 synchronized worms per dilution (RNAeasy; Qiagen, http://www1.qiagen.com). When vector-only–treated animals had become 24-h-old gravid adults, all RNAi dilutions were collected and processed as previously described [15]. Unique primer pairs recognizing only cDNA derived from endogenous mRNA were designed to avoid cross-reaction with genomic DNA and bacterially generated dsRNA that invoked the RNAi effect (for *atp-3*). The primer pairs used are as follows: *atp-3* ForC: 5′ ACGGTGACTTATGCCGTCAAG 3′, *atp-3* RevB: 5′ TTAGATGGCGGTGGCAAGG 3′; and (for normalization purposes) *ama-1* For: 5′ GCGGTCAGAAAGGCTATCGA 3′, *ama-1* Rev: 5′ AGCAGTGCCAAATGTCGGTAAT 3′.

### Western blot analysis.

Whole-worm lysates were prepared from approximately 200 animals in boiling 5% SDS, 0,02% β-mercaptoethanol, and 1 mM protease inhibitor cocktail (SIGMA P2714; Sigma, http://www.sigmaaldrich.com). Following protein quantitation (BCA; Pierce Biotechnology, http://www.piercenet.com), protein levels were measured by western analysis, quantified using densitometry, then normalized against actin. Antibodies employed were anti-NUO-2 (MS112, 1:2,000 dilution; MitoSciences, http://www.mitosciences.com), anti-Actin (AC-15, 1:5,000 dilution; Sigma), anti-ANT (MSA02, 1:1,000 dilution; MitoSciences), anti-complex IV, subunit I (MS404, 1:1,000 dilution; MitoSciences), and anti-complex V, α-subunit (MS507, 1:1,000 dilution; MitoSciences).

### Protein oxidation.

Protein oxidation in whole-worm extracts were determined using the Oxyblot method (Chemicon, http://www.chemicon.com) and western analysis with a 3-nitrotyrosine antibody (Upstate Biotechnology, http://www.upstate.com). In the Oxyblot method, 2,4-dinitrophenylhydrazine (DNP) reacts to form stable adducts with carbonyl groups, to act as a surrogate marker for nonspecific protein oxidation. The resulting adducts can be detected using an anti-DNP antibody in a standard western assay. For both methods, total oxidized protein was quantified using densitometry and NIH ImageJ (v1.36b). Differences in protein loading were normalized based on Ponceau staining.

### Size determination.

RNAi dilution series were established in an N2 background. For *atp-3* and *nuo-2* (*n* = 500 and 200, respectively), when vector-only–treated animals had become 24-h-old gravid adults, all RNAi dilutions were collected and sized using a COPAS Biosort 250 Worm Sorter (Union Biometrica, Harvard Biosciences, http://www.unionbio.com). For all other dilution series, individual animals were randomly selected, then arranged for image analysis on 2% agarose pads.

### Microscopy.

Nomarski images were collected using a PCO SensiCam charge-coupled diode camera (http://www.pco.de) connected to a Zeiss Axioskop running SlideMaker 4.0 software (Carl Zeiss, http://www.zeiss.com).

### Data access.

Raw data used in the construction of figures are available upon request (SLR).

## Supporting Information

Figure S1 Decomposition of Mean Lifespan Expectancy Data in Figure 1
Individual *atp-3* and *nuo-2* lifespan experiments used to construct Figure 1 are shown (red lines).
(2.0 MB TIF)

Figure S2 Decomposition of Mean Lifespan Expectancy Data in Figure 2A–2C Individual *cco-, isp-1,* and *nuo-2* lifespan experiments used to construct Figure 2A–2C are shown.
(1.8 MB TIF)

Figure S3 Survival Analysis of *isp-1(gk267)*
The strain VC520 was employed to assess the longevity of homozygous *isp-1(gk267)* animals. Shown is a survival plot illustrating the increased longevity of homozygous *isp-1(gk267*) relative to balanced (control) animals. Note that homozygous *isp-1(gk267)* animals arrest as early larvae (*n* = 60 worms/condition).
(898 KB TIF)

Figure S4 Additional Mit Mutant Dilution Series RNAi dilution series for *atp-3, D2030.4, tag-55, F26E4.6,* and *W09C5.8* were established using N2 animals (left column). In some cases, *eri-1(mg366)* (middle column) and *rrf-3(pk1426)* (right column) mutant strains were also employed. For each experiment, when vector-only–treated animals became 1-d-old gravid adults, individual worms were randomly selected from all RNAi dilutions and then collectively mounted for Normaski imaging (400× magnification, left to right, target gene to empty vector ratio: 0:1, 1:1,000, 1:750, 1:500, 1:200, 1:100, 1:50, 1:20, 1:10, 1:5, 1:2, and 1:0. The following dilutions are absent from the *atp-3* dilution series: 1:750, 1:200, and 1:20. Refer to Figure 1A for a complete *atp-3* dataset). Scale bar indicates 100 μm.
(1.9 MB TIF)

Figure S5 Undiluted *mev-1* RNAi Blocks Adult Growth and Leads to Infertility Synchronously laid N2 animals were fed bacteria containing either a *mev-1* RNAi feeding construct (bottom row) or vector control (top row) from the time of hatching. After 3 d, both populations of animals had reached adulthood, but there was a noticeable, although slight, reduction in adult size. At later times, it became evident that *mev-1* animals failed to undergo adult growth (days 4 and 6). There was a substantial reduction in the number of eggs produced by undiluted *mev-1* RNAi–fed animals, all of which failed to hatch (right column: shown are progeny from day 4 adults photographed on day 6). Magnification varies across rows but is identical in each column.
(7.3 MB TIF)

Table S1 *atp-3* RNAi Dilution Series: Larval Development Rates (98 KB DOC)

Table S2 Overlapping Roles of Genes Associated with Mit Mutant Longevity, Embryonic Mitosis, and Transposon Silencing (286 KB DOC)

### Accession Numbers

The Wormbase (http://www.wormbase.org) accession numbers for the genes and gene products discussed in the paper are as follows: *atp-3* (WBGene00000230), *cco-1* (WBGene00000371), *cyc-1* (WBGene00000869), *frh-1* (WBGene00001486), *isp-1* (WBGene00002162), *mev-1* (WBGene00003225), *nuo-2* (WBGene00020417), and SKN-1 (WBGene00004804).

## Abbreviations

ANT: adenine nucleotide transporter
AMPK: adenosine monophosphate (AMP)–activated protein kinase
ATP: adenosine triphosphate
ETC: electron transport chain
L: larval
mtDNA: mitochondrial DNA
NADH: nicotinamide adenine dinucleotide
RNAi: RNA interference
ROS: reactive oxygen species
SEM: standard error of the mean
