# Correction: Relationship Between Mitochondrial Electron Transport Chain Dysfunction, Development, and Life Extension in Caenorhabditis elegan

**DOI:** 10.1371/journal.pbio.0060023

**Published:** 2008-01-29

**Authors:** Shane L Rea, Natascia Ventura, Thomas E Johnson

Correction for:

Rea SL, Ventura N, Johnson TE (2007) Relationship between mitochondrial electron transport chain dysfunction, development, and life extension in Caenorhabditis elegans. PLoS Biol 5(10): e259. doi:10.1371/journal.pbio.0050259

The corresponding author's e-mail is incorrect. It should be: reas3@uthscsa.edu.

