# Correction: Relationship Between Mitochondrial Electron Transport Chain Dysfunction, Development, and Life Extension in Caenorhabditis elegans

**DOI:** 10.1371/journal.pbio.0060078

**Published:** 2008-03-25

**Authors:** Shane L Rea, Natascia Ventura, Thomas E Johnson

Correction for:

Rea SL, Ventura N, Johnson TE (2007) Relationship between mitochondrial electron transport chain dysfunction, development, and life extension in Caenorhabditis elegans. PLoS Biol 5(10): e259. doi:10.1371/journal.pbio.0050259

The author affiliations were incorrect. The correct affiliations should appear as below:

**Shane L. Rea^1,2
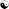
*^, Natascia Ventura^1,3
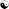
^, Thomas E. Johnson^1^**

**1** Institute for Behavioral Genetics, University of Colorado at Boulder, Boulder, Colorado, United States of America,

**2** Sam and Ann Barshop Institute for Longevity and Aging Studies and the Department of Physiology, University of Texas Health Science Center at San Antonio, San Antonio, Texas, United States of America,

**3** Laboratory of Signal Transduction, Department of Experimental Medicine and Biochemical Sciences, University of Rome “Tor Vergata”, Rome, Italy

* To whom correspondence should be addressed. E-mail: reas3@uthscsa.edu


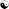
These authors contributed equally to this work.

Also, there was an omission in the funding information. The correct funding line is: Financial support was provided by the National Institute on Aging (SLR R21 AG025207 and TEJ RO1 AG16219), the Polis Foundation (SLR and TEJ), the Ellison Medical Foundation (TEJ), the National Ataxia Foundation (NV), and the Italian Federation for Cancer Research (FIRC) (NV).

