# Supplementary material for: Relationship Between Mitochondrial Electron Transport Chain Dysfunction, Development, and Life Extension in Caenorhabditis elegans
Source: PLoS Biol. 2007 Oct 2;5(10):e259. doi: 10.1371/journal.pbio.0050259 (PMC1994989; doi:10.1371/journal.pbio.0050259)
Supplement: Supplementary file 1 [file pbio.0050259.st001.doc]

Supplementary Table I. *atp-3* RNAi Dilution Series: Larval Development Rates.

| **Time (hr)*** | **Vector** | **1 in 1000** | **1 in 750** | **1 in 500** | **1 in 200** | **1 in 100** | **1 in 50** | **1 in 20** | **1 in 10** | **1 in 5** | **1 in 2** | **Undiluted** |
| --- | --- | --- | --- | --- | --- | --- | --- | --- | --- | --- | --- | --- |
| **0** | Egg Lay | Egg Lay | Egg Lay | Egg Lay | Egg Lay | Egg Lay | Egg Lay | Egg Lay | Egg Lay | Egg Lay | Egg Lay | Egg Lay |
| **15** | Hatch | Hatch | Hatch | Hatch | Hatch | Hatch | Hatch | Hatch | Hatch | Hatch | Hatch | Hatch |
| **23** | Mid L1** | Mid L1 | Mid L1 | Mid L1 | Mid L1 | Mid L1 | Mid L1 | Mid L1 | Mid L1 | Mid L1 | Mid L1 | Mid L1 |
| **29** | Late L2 (100%)^†^ | Late L2 (100%) | Late L2 (100%) | Late L2 (100%) | Early L2 (3%)  Late L2 (97%) | Late L2 (100%) | Late L2 (100%) | Early L2 (3%)  Late L2 (97%) | Late L2 (100%) | Early L2 (3%)  MId L2 (97%) | Early L2 (100%) | Early L2 (100%) |
| **41** | Late L3 (97%)  Early L4 (3%) | Mid L3 (37%)  Late L3 (63%) | Early L3 (40%)  Mid L3 (57%)  Late L3 (3%) | Early L3 (37%)  Mid L3 (50%)  Late L3 (13%) | Mid L3 (100%) | Mid L3 (100%) | Early L3 (100%) | Early L3 (100%) | Early L3 (100%) | Late L2 (4%)  Early L3 (96%) | Late L2 (100%) | Late L2 (100%) |
| **48** | Late L4 (100%) | Late L4 (97%)  YA (3%) | Late L4 (100%) | Mid-Late L4 (100%) | Mid-Late L4 (100%) | Mid-Late L4 (93%)  YA (7%) | Early L4 (50%)  Mid L4 (50%) | Early L4 (36%)  Mid L4 (61%)  Late L4(3%) | Early L4 (100%) | Late L2 (4%)  Mid L3 (96%) | Early L3 (100%) | Late L2 (4%)  Early L3 (96%) |
| **58** | YA (70%)  GA (30%) | YA (57%)  GA (43%) | YA (66%)  GA (33%) | YA (63%)  GA (47%) | YA (60%)  GA (40%) | YA (57%)  GA (43%) | Late L4 (9%)  YA (97%) | Late L4 (68%)  YA (32%) | Mid-Late L4 (100%) | Mid L3 (100%) | Early L3 (100%) | Late L2 (4%)  Early L3 (96%) |
| **84** | GA (100%)^††^ | GA (100%)^††^ | GA (100%)^††^ | GA (100%)^††^ | GA (100%)^††^ | GA (100%)^††^ | GA (100%)^††^ | YA (82%)  GA (18%) | Late L4 (17%)  YA (83%) | Mid L3 (4%)  Mid L4 (96%) | Mid L3 (96%)  Early L4 (4%) | Late L2 (4%)  Mid L3 (96%) |
| **105** |  |  |  |  |  |  |  | GA (100%)^††^ | Late L4 (7%)  Ste Adult (70%)  GA (23%) | Late L3 (5%)  Late L4 (13%)  Ste Adult (82%) | Late L3 (88%)  Late L4 (6%)  Ste Adult (6%) | Mid L3 (100%) |
| **130** |  |  |  |  |  |  |  |  | Ste Adult (100%)^††^ | Late L3 (5%)  Late L4 (9%)  Ste Adult (81%)  Dead (5%) | Early L4 (56%) Late L4 (38%)  Ste Adult (6%) | Early L4 (75%) Late L4 (17%) Dead (8%) |
| **156** |  |  |  |  |  |  |  |  |  | Late L3 (5%)  Ste Adult (95%) | Early L4 (7%)  Late L4 (67%)  Ste Adult (20%) Dead (7%) | Early L4 (20%) Late L4 (60%)  Ste Adult (10%)  Dead (10%) |
| **182** |  |  |  |  |  |  |  |  |  | Early L4 (5%)  Ste Adult (90%)  Dead (5%)^††^ | Early L4 (7%)  Late L4 (23%)  Ste Adult (38%) Dead (31%) | Late L4 (63%)  Ste Adult (12%)  Dead (25%) |
| **207** |  |  |  |  |  |  |  |  |  |  | Late L4 (30%)  Ste Adult (50%) Dead (20%)^††^ | Late L4 (12%) Dead (88%)^††^ |

* Time zero was taken as the time at which eggs were laid (20°C).

** L1 – L4, first to fourth larval stages; YA, young adult (pre-egg laying); GA, gravid adult (egg-laying); Ste Adult, sterile adult (non-egg producing);

Dead, observed death (bagged, vulva-prolapsed, dessicated and lost individuals were excluded from analysis).

^†^ Numbers in parentheses, percentage of population at the indicated developmental stage.

^††^ Scoring was halted after this point.
