# Supplementary material for: Relationship Between Mitochondrial Electron Transport Chain Dysfunction, Development, and Life Extension in Caenorhabditis elegans
Source: PLoS Biol. 2007 Oct 2;5(10):e259. doi: 10.1371/journal.pbio.0050259 (PMC1994989; doi:10.1371/journal.pbio.0050259)
Supplement: Supplementary file 2 [file pbio.0050259.st002.doc]

Supplementary Table II. Overlapping Roles of Genes Associated with Mit Mutant Longevity, Embryonic Mitosis and Transposon Silencing.

|  |  | **Phenotype** |  |  |
| --- | --- | --- | --- | --- |
| **Gene *** | **Life Extension **** | **Defective Mitosis ^†^** | **Loss of Transposon Silencing ^‡^** | **Notes ^‡‡^** |
| **Mitochondria** |  |  |  |  |
| **ETC** |  |  |  |  |
| **Complex I** |  |  |  |  |
| *nuo-1(ua1)* | ✓ (G) ^[1]^ |  |  | NDUFV1, (C09H10.3), arrest as L3 |
| *nuo-2* | ✓ (R) ^[2,3]^ | ✓ (General Pace of Development Slowed) |  | NDUFS3, (T10E9.7) |
| *nuo-3* | ✓ (R) ^[2]^ |  |  | NDUFA6, (Y57G11C.12) |
| *nuo-4* | ✓ (R) ^[2,4]^ | ✓ (General Pace of Development Slowed) |  | NDUFA10, (K04G7.4) |
| *nuo-5* | ✓ (R) ^[2]^ |  |  | NDUFS1, (Y45G12B.1) |
| D2030.4 | ✓ (R) ^[4,5]^ | ✓ (General Pace of Development Slowed) |  | NDUFB7 |
| T20H4.5 | ✓ (R) ^[4]^ | ✓ (General Pace of Development Slowed) |  | NDUFS8 |
| C25H3.9 |  | ✓ (General Pace of Development Slowed) |  | NDUFB5 |
| F44G4.2 |  | ✓ (General Pace of Development Slowed) |  | NDUFB2 |
| F59C6.5 |  | ✓ (General Pace of Development Slowed) |  | NDUFB10 |
| W10D5.2 |  | ✓ (General Pace of Development Slowed) |  | NDUFS7 |
|  |  |  |  |  |
| **Complex II** |  |  |  |  |
| *mev-1* |  | ✓ (General Pace of Development Slowed) |  | SDHC, (T07C4.7) |
| C03G5.1 |  | ✓ (Polar Body Extrusion Abnormal) |  | SDHA, (C03G5.1) |
|  |  |  |  |  |
| **Complex III** |  |  |  |  |
| *cyc-1* | ✓ (R) ^[2,3]^ |  |  | cytochrome c_1,_ (C54G4.8) |
| *isp-1(qm150)* | ✓ (G, R) ^[6],^ **^††^** |  |  | Rieske Fe-S protein, (F426G8.12 ) |
| T02H6.11 | ✓ (R ^§^) ^[5]^ |  |  | UQCRB |
| T24C4.1 |  |  | ✓ | UQCRC2 |
|  |  |  |  |  |
| **Complex IV** |  |  |  |  |
| *cco-1* | ✓ (R) ^[2,3,4,5]^ |  |  | COX5B, (F26E4.9) |
| *cco-2* | ✓ (R) ^[2]^ |  |  | COX5A, (Y37D8A.14) |
| F26E4.6 | ✓ (R) ^[4,5]^ |  |  | COX7C |
| F54D8.2 | ✓ (R ^§^) |  |  | COX6A1 |
| W09C5.8 | ✓ (R) ^[4,5]^ |  |  | COX4I1 |
|  |  |  |  |  |
|  |  |  |  |  |
|  |  |  |  |  |
| **Supplementary Table II.** (*continued* ) | | |  |  |
|  |  | **Phenotype** |  |  |
| **Gene *** | **Life Extension **** | **Defective Mitosis ^†^** | **Loss of Transposon Silencing ^‡^** | **Notes ^‡‡^** |
| **Mitochondria** |  |  |  |  |
| **ETC** |  |  |  |  |
| **Complex V** |  |  |  |  |
| *asg-1* |  | ✓ (General Pace of Development Slowed) ^§§^ | ✓ ^§§^ | ATP5L, (K07A12.3), 98.5% similar to *asg-2* |
| *asg-2* | ✓ (R ^§§^) ^[4]^ |  |  | ATP5L, (C53B7.4), 98.5% similar to *asg-1* |
| *asb-1* |  | ✓ (General Pace of Development Slowed) ^§§, 8^ | ✓ ^§§^ | ATP5F1 (F35G12.10), 99% similar to *asb-2* |
| *asb-2* | ✓ (R ^§§^) ^[2]^ |  |  | ATP5F1, (F02E8.1), 99% similar to *asb-1* |
| *atp-2(ua2)* | ✓ (G) ^[1]^ | ✓ (General Pace of Development Slowed) |  | ATP5B, (C34E10.6), catalytic subunit, arrest as L3 |
| *atp-3* | ✓ (R) ^[2,3]^ | ✓ (Osmotic Integrity of Egg Compromised) |  | ATP5O, (F27C1.7) |
| *atp-4* | ✓ (R) ^[2]^ | ✓ (General Pace of Development Slowed) |  | ATP5J, (T05H4.12) |
| *atp-5* | ✓ (R) ^[2]^ | ✓ (General Pace of Development Slowed) |  | ATP5H, (C06H2.1) |
| F58F12.1 |  | ✓ (General Pace of Development Slowed) |  | ATP5D |
| H28O16.1 |  | ✓ (General Pace of Development Slowed) |  | ATP5A1 |
| R54.3 |  | ✓ (General Pace of Development Slowed) |  | ATP5J2 |
| Y69A2AR.18 |  | ✓ (General Pace of Development Slowed) |  | ATP5C1 |
| Y82E9BR.3 |  | ✓ (General Pace of Development Slowed) |  | ATP5G3 |
| **Other ETC** ^¶^ |  |  |  |  |
| *cchl-1* | ✓ (R ^§^) ^[2,5]^ |  |  | Holocytochrome c synthase/heme-lyase, (T06D8.6) |
| *clk-1(qm30, e2519)* | ✓ (G) ^[7]^ | ✓ ^[7]^ |  | CLK-1, (ZC395.2), UQ biosynthesis, maternal effect |
| *ers-2* |  | ✓ (Severe Pleiotropic Defects) |  | Mitochondrial glutamyl-tRNA synthetase, (ZC434.5) |
| *frh-1* | ✓ (G, R) ^[8]^ |  |  | Frataxin, (F59G1.7), defective Fe-S cluster assembly |
| *gro-1(e2400)* | ✓ (G) ^[9]^ |  |  | tRNA δ(2)-isopentenylpyrophosphate transferase, (ZC395.6), maternal effect |
| *lrs-2(mg312)* | ✓ (G) ^[5]^ | ✓ (Severe Pleiotropic Defects) |  | Leucyl-tRNA synthetase, (ZK524.3), maternal effect |
| *qrs-5* |  | ✓ (Severe Pleiotropic Defects) |  | Mitochondrial glutamyl-tRNA synthetase, (Y41E3.4) |
| *tufm-1* |  |  | ✓ | Mitochondrial translation elongation factor Tu (Y71H2AM.23) |
| B0261.4 | ✓ (R) ^[4,5]^ |  |  | Mitochondrial 39S ribosomal protein L47 |
| C08F8.2 |  |  | ✓ | RNA helicase (SUV3), DEAD-box superfamily |
| C41G7.3 |  | ✓ (General Pace of Development Slowed) |  | Peripheral-type benzodiazapine receptor |
| F25B4.6 |  | ✓ (Osmotic Integrity) |  | HMG-CoA synthase, necessary for UQ synthesis |
| T01B11.4 |  | ✓ (General Pace of Development Slowed) |  | ANT1, ADP/ATP carrier, worms have 7 ANT homologues |
| T27E9.1 | ✓ (R ^§^) | ✓ (General Pace of Development Slowed) |  | ANT2, ADP/ATP carrier, worms have 7 ANT homologues |
|  |  |  |  |  |
| **Supplementary Table II.** (*continued* ) | | |  |  |
|  |  | **Phenotype** |  |  |
| **Gene *** | **Life Extension **** | **Defective Mitosis ^†^** | **Loss of Transposon Silencing ^‡^** | **Notes ^‡‡^** |
| **Mitochondria** |  |  |  |  |
| **TCA cycle ^¶¶^** |  |  |  |  |
| *aco-2* | ✓ (R) ^[4]^ | ✓ (General Pace of Development Slowed) | ✓ | Aconitase (F54H12.1) |
| *cts-1* |  | ✓ (General Pace of Development Slowed) |  | Citrate synthase, (T20G5.2) |
| *tpk-1(qm162)* | ✓ (G) ^[10]^ |  |  | Thiamine pyrophosphokinase, (ZK637.9), (pyruvate dehydrogenase, α-ketoglutarate dehydrogenase and branched chain keto-acids dehydrogenases affected) |
| F43G9.1 | ✓ (R) ^[4]^ |  | ✓ | Isocitrate dehydrogenase, α subunit (NAD^+^-specific) |
| F35G12.2 |  | ✓ (General Pace of Development Slowed) |  | Isocitrate dehydrogenase, γ subunit (NAD^+^-specific) |
| F23B12.5 |  | ✓ (General Pace of Development Slowed) |  | Dihydrolipoamide acetyltransferase, (pyruvate dehydrogenase, α-ketoglutarate dehydrogenase and branched chain keto-acids dehydrogenases affected) |
| M01F1.3 |  |  | ✓ | Lipoic acid synthetase |
| T05H10.6 |  | ✓ (General Pace of Development Slowed) |  | Pyruvate dehydrogenase, α subunit |
| T22B11.5 |  | ✓ (General Pace of Development Slowed) |  | α-ketoglutarate dehydrogenase, E1 subunit |
| W02F12.5 |  | ✓ (Severe Pleiotropic Defects) |  | α-ketoglutarate dehydrogenase, E2 subunit, (dihydrolipoamide succinyltransferase) |
| **TCA Substrate**  **Transfer** |  |  |  |  |
| C33F10.12 | ✓ (R) ^[4]^ |  |  | Mitochondrial phosphate carrier protein |
| F13G3.7 | ✓ (R) ^[5]^ |  |  | Mitochondrial carrier protein |
| K01C8.7 | ✓ (R) ^[5]^ |  |  | Mitochondrial FAD carrier protein |
| Y37B11A.3 |  | ✓ (Complex Phenotype) |  | Tricarboxylate/dicarboxylate carrier protein |
| F59B8.2 | ✓ (R) ^[4]^ |  |  | Isocitrate dehydrogenase (NADP^+^-dependent), cytoplasmic |
| **Protein Import**  **Machinery** |  |  |  |  |
| *tim-17* |  | ✓ (General Pace of Development Slowed) |  | Inner mitochondrial membrane translocase, (E04A4.5) |
| *tim-22* |  | ✓ (Osmotic Integrity) |  | Inner mitochondrial membrane translocase, (C47G2.3) |
| *tim23* |  | ✓ (Severe Pleiotropic Defects) |  | Inner mitochondrial membrane translocase, (F15D3.7) |
| *tim-44* |  | ✓ (General Pace of Development Slowed) | ✓ | Inner Mitochondrial Membrane Translocase, (T09B4.9) |
| *tom22* |  | ✓ (General Pace of Development Slowed) |  | Outer Mitochondrial Membrane Translocase, (W10D9.5) |
|  |  |  |  |  |
|  |  |  |  |  |
|  |  |  |  |  |
| **Supplementary Table II.** (*continued* ) | | |  |  |
|  |  | **Phenotype** |  |  |
| **Gene *** | **Life Extension **** | **Defective Mitosis ^†^** | **Loss of Transposon Silencing ^‡^** | **Notes ^‡‡^** |
| **Mitochondria** |  |  |  |  |
| **Protein Folding** |  |  |  |  |
| *hsp-6* | ✓ (R) ^[11]^ | ✓ (General Pace of Development Slowed) |  | Heat shock protein-6, (C37H5.8) |
| *phb-1* |  | ✓ (General Pace of Development Slowed) ^¶¶¶^ |  | Prohibitin-1, (Y37E3.9), dimerizes with PHB-2 |
| *phb-2* |  |  | ✓ ^¶¶¶^ | Prohibitin-2, (T24H7.1), dimerizes with PHB-1 |
| F56D2.1 |  | ✓ (General Pace of Development Slowed) |  | Processing peptidase, β subunit |
|  |  |  |  |  |
| **Cytoplasmic** |  |  |  |  |
| **Glycolysis** |  |  |  |  |
| F57B10.3 | ✓ (R) ^[4,5]^ |  |  | Phosphoglycerate mutase |
| F01F1.12 |  | ✓ (General Pace of Development Slowed) |  | Fructose-bisphosphate aldolase |
|  |  |  |  |  |
| **Function Unknown** |  |  |  |  |
| *ril-1* | ✓ (R) ^[2]^ | ✓ (General Pace of Development Slowed) |  | RNAi-induced longevity-1, (C53A5.1) |
|  |  |  |  |  |

* Genes are categorized according to subcellular locality and function. Where applicable, specific alleles are described in parenthesis. Blue colour indicates Mit mutant associated with defective embryonic mitosis. Red colour indicates Mit mutant associated with both defective embryonic mitosis and transposon desilencing. ** In parenthesis, RNAi-induced (R) or Genetically-defined (G) Mit mutant. ^†^ All genes defined by Sonnichsen and colleagues [12], except where indicated. The phenotypic class to which each mutant belongs (as defined in Table I of [12]]), is indicted in parenthesis. ^††^ This study. ^‡^ All genes identified by Vastenhouw and colleagues [13]]; ^‡‡^ Human protein orthologue listed first. Where relevant, unique cosmid identifier listed in parenthesis. Additional notes follow. (Refer to Tsang and Lemire [14]], for a complete description of gene orthology and ETC subunit nomenclature). ^§^ In the screen of Hamilton and colleagues [4]], this gene only extended the longevity of N2 and not *rrf-3(pk1426)* under the tested conditions (S.S. Lee, *personal communication*). ^§§^ RNAi used in this screen cross-reacted with paralogoue. ^¶^ Ribosomal, tRNA and DNA replication genes are included since the only polypeptides encoded by mitochondrial DNA are ETC subunits. ^¶¶^ Genes encoding enzymes necessary for the synthesis of co-factors for TCA cycle enzyme genes are also included in this category. ^¶¶¶^ PHB-1 and PHB-2 heterodimerize.

**Supplementary References:**

1. Tsang WY, Sayles LC, Grad LI, Pilgrim DB, Lemire BD (2001) Mitochondrial Respiratory Chain Deficiency in Caenorhabditis elegans Results in Developmental Arrest and Increased Life Span. J Biol Chem 276: 32240-32246.

2. Hansen M, Hsu A-L, Dillin A, Kenyon C (2005) New Genes Tied to Endocrine, Metabolic, and Dietary Regulation of Lifespan from a Caenorhabditis elegans Genomic RNAi Screen. PLoS Genetics 1: e17.

3. Dillin A, Hsu A-L, Arantes-Oliveira N, Lehrer-Graiwer J, Hsin H, et al. (2002) Rates of Behavior and Aging Specified by Mitochondrial Function During Development. Science 298: 2398-2401.

4. Hamilton B, Dong Y, Shindo M, Liu W, Odell I, et al. (2005) A systematic RNAi screen for longevity genes in C. elegans. Genes Dev 19: 1544-1555.

5. Lee SS, Lee RY, Fraser AG, Kamath RS, Ahringer J, et al. (2003) A systematic RNAi screen identifies a critical role for mitochondria in C. elegans longevity. Nat Genet 33: 40-48.

6. Feng J, Bussière F, Hekimi S (2001) Mitochondrial Electron Transport Is a Key Determinant of Life Span in Caenorhabditis elegans. Developmental Cell 1: 633-644.

7. Colaiacovo MP, Stanfield GM, Reddy KC, Reinke V, Kim SK, et al. (2002) A Targeted RNAi Screen for Genes Involved in Chromosome Morphogenesis and Nuclear Organization in the Caenorhabditis elegans Germline. Genetics 162: 113-128.

8. Ventura N, Rea S, Henderson ST, Condo I, Johnson TE, et al. (2005) Reduced expression of frataxin extends the lifespan of Caenorhabditis elegans. Aging Cell 4: 109-112.

9. Lakowski B, Hekimi S (1996) Determination of life-span in Caenorhabditis elegans by four clock genes. Science 272: 1010-1013.

10. de Jong L, Meng Y, Dent J, Hekimi S (2004) Thiamine Pyrophosphate Biosynthesis and Transport in the Nematode Caenorhabditis elegans. Genetics 168: 845-854.

11. Ventura N, Rea SL (2007) *Caenorhabditis elegans* mitochondrial mutants as an investigative tool to study human neurodegenerative diseases associated with mitochondrial dysfunction. Biotechnology Journal 9999: NA.

12. Sonnichsen B, Koski LB, Walsh A, Marschall P, Neumann B, et al. (2005) Full-genome RNAi profiling of early embryogenesis in Caenorhabditis elegans. 434: 462-469.

13. Vastenhouw NL, Fischer SEJ, Robert VJP, Thijssen KL, Fraser AG, et al. (2003) A Genome-Wide Screen Identifies 27 Genes Involved in Transposon Silencing in C. elegans. Current Biology 13: 1311-1316.

14. Tsang WY, Lemire BD (2003) The role of mitochondria in the life of the nematode, Caenorhabditis elegans. Biochimica et Biophysica Acta (BBA) - Molecular Basis of Disease 1638: 91-105.
